# Supplementary material for: Chitinolytic and Fungicidal Potential of the Marine Bacterial Strains Habituating Pacific Ocean Regions
Source: Microorganisms. 2023 Sep 8;11(9):2255. doi: 10.3390/microorganisms11092255 (PMC10535946; doi:10.3390/microorganisms11092255)
Supplement: Supplementary file 1 [file microorganisms-11-02255-s001.zip › microorganisms-2586383-Supplementary Tables.pdf]

Table S1. A list of bacterial strains with chitinolytic phenotypes

№	Strain Number	Taxonomic Affiliation
1	M-3Alg 48/3w	*Zobellia galactanivorans*
2	M-5Alg 13/2	*Vibrio* sp.
3	6 Alg 5/1	*Aquimarina* sp.
4	6 Alg 69.	Unknown
5	6 Alg 70	Unknown
6	6 Alg 71	Unknown
7	6 Alg 104	*Aquimarina aggregata*
8	6Alg 185	*Aquimarina* sp.
9	9 Alg 16 Prote	*Vibrio* sp.
10	9Alg 45	*Vibrio jasicida*
11	9 Alg 79/2	*Aquimarina algiphila*
12	9 Alg 80.	*Aquimarina algiphila*
13	9 Alg 134	Unknown
14	9 Alg 152	*Aquimarina algiphila*
15	9Alg 297	*Pseudoalteromonas arabiensis*
16	M-Scm 11	Unknown
17	M-Sg 44^{T}	*Arenicella chitinivorans*
18	M-Sgf 3	*Vibrio* sp.
19	M-Sgf 5	Unknown
20	M-Sgf 11	Unknown
21	M-Sgf 18	Unknown
22	Sgf 25	*Microbulbifer thermotolerans*
23	M-Sgm 5	*Vibrio* sp.
24	M-Sgm 13	Unknown
25	M-Sgm 15	Unknown
26	M-Sgm 22	*Vibrio* sp.
27	M-Sgm 24	Unknown
28	M-Sgm 25/1	*Microbulbifer thermotolerans*
29	M-Sgm25/4	Unknown
30	M-Sgm 27	Unknown
31	M-Sgm 28	Unknown
32	M-Sgm28/2	Unknown
33	Sh 1	*Microbulbifer thermotolerans*
34	M-Sh 2	*Microbulbifer thermotolerans*
35	M-Sh 3	*Microbulbifer thermotolerans*
36	M-Sh 4	*Microbulbifer thermotolerans*
37	M-Sh 5	*Microbulbifer thermotolerans*
38	M-Sh 72/1^{T}	*Echinimonas agarilytica*
39	M-Sh 72/3	Unknown

40	M-Sh 82	Unknown
41	IDSW-1	*Pseudoalteromonas* sp.
42	IDSW-36	*Vibrio pomeroyi*
43	IDSW-37	Unknown
44	IDSW-38.	Unknown
45	V1SW 45	*Aquimarina muelleri*
46	V1SW 49^T	*Aquimarina muelleri*
47	V1SW 51	*Aquimarina muelleri*
48	V1SW 55	*Aquimarina muelleri*
49	30-Bs-8	Unknown
50	30-Bs-7	Unknown
51	M-14G-20	*Vibrio* sp.
52	M-14G-21	*Microbulbifer thermotolerans*
53	M-14G-22	*Microbulbifer thermotolerans*
54	NITRA-45	*Vibrio parahaemolyticus*
55	KCTC	*Microbulbifer salipaludis*
56	JCM 14709^T	*Microbulbifer thermotolerans*
57	KMM 3630^T	*Peudoalteromonas flavipulchra*
58	NCIMB 2128^T	*Peudoalteromonas undina*
59	CIP 75.02^T	*Vibrio parahaemolyticus*
60	B 530	*Pseudoalteromonas* sp.
61	B 532/1	*Serratia* sp.
62	B 532/2 pink	*Serratia* sp.
63	B 737	Unknown
64	B 739	Unknown
65	B 828	Unknown
66	Dji^T	*Zobellia galactanivorans*

Table S2. The genomes IDs of the analysed chitinolytic strains deposited in GenBank/EMBL/DDBJ

BioProject	BioSample	Accession	Organism
PRJNA898858	SAMN31633994	JAPHPV000000000	*Pseudoalteromonas* sp.B530 (KMM6841)
PRJNA898858	SAMN31633993	JAPHPW000000000	*Vibrio* sp. 14G-20 (KMM6839)
PRJNA898858	SAMN31633992	JAPHPX000000000	*Vibrio* sp. Sgm 22 (KMM6833)
PRJNA898858	SAMN31633991	JAPHPY000000000	*Vibrio* sp. Sgm 5 (KMM6832)
PRJNA898858	SAMN31633990	JAPHPZ000000000	*Aquimarina muelleri* V1SW51 (KMM6556)
PRJNA898858	SAMN31633989	JAPHQA000000000	*M. thermotolerans* 14G-22 (KMM6840)

PRJNA898858	SAMN31633988	JAPHQB000000000	*M. thermotolerans* Sh5 (KMM6838)
PRJNA898858	SAMN31633987	JAPHQC000000000	*M. thermotolerans* Sh4 (KMM6837)
PRJNA898858	SAMN31633986	JAPHQD000000000	*M. thermotolerans* Sh3 (KMM6836)
PRJNA898858	SAMN31633985	JAPHQE000000000	*M. thermotolerans* Sh2 (KMM 6835)
PRJNA898858	SAMN31633984	JAPHQF000000000	*M. thermotolerans* Sh1 (KMM 6242)
PRJNA898858	SAMN31633983	JAPHQG000000000	*M. thermotolerans* Sgm 25/1 (KMM 6834)
PRJNA898858	SAMN31633982	JAPHQH000000000	*M. thermotolerans* Sgf 25 (KMM 6262)

Table S3. Comparative characteristics of the marine chitinolytic bacteria genomes.

Accession	Genes (Total)	CDSs (Total)	Genes (Coding)	CDSs (with Protein)	Genes (RNA)	rRNAs	Complete rRNAs	tRNAs	ncRNAs	Pseudo Genes (Total)	CDSs (without Protein)	GC Content	Contig N50	Contig L50
Pseudoalteromonas sp,B530 (KMM 6841)														
JAPHPV000000000	4,720	4,632	4,602	4,602	97	6, 5, 4 (5S, 16S, 23S)	3 (5S)	78	4	30	30	43,1	213040	8
Vibrio sp, 14G-20 (KMM 6839)														
JAPHPW000000000	4,504	4,431	4,381	4,381	73	2, 3, 5 (5S, 16S, 23S)	1 (5S)	59	4	50	50	44,3	200141	10
Vibrio sp, Sgm22 (KMM 6833)														
JAPHPX000000000	4,528	4,430	4,380	4,380	98	3, 7, 5 (5S, 16S, 23S)	1 (5S)	79	4	50	50	44,4	228413	9
Vibrio sp, Sgm5 (KMM 6832)														
JAPHPY000000000	5,315	5,225	5,142	5,142	90	2, 5, 2 (5S, 16S, 23S)	2, 1 (5S, 16S)	77	4	83	83	45,0	93443	21
Aquimarina muelleri V1SW51 (KMM 6556)														
JAPHPZ000000000	4,152	4,107	4,079	4,079	45	1, 1, 1 (5S, 16S, 23S)	1, 1, 1 (5S, 16S, 23S)	38	4	28	28	31,4	83426	18
M. thermotolerans 14G-22 (KMM 6840)														
JAPHQA000000000	3,375	3,324	3,289	3,289	51	1, 1, 1 (5S, 16S, 23S)	1, 1, 1 (5S, 16S, 23S)	44	4	35	35	56,5	153113	9
M. thermotolerans Sh5 (KMM6838)														
JAPHQB000000000	3,382	3,331	3,289	3,289	51	1, 1, 1 (5S, 16S, 23S)	1, 1, 1 (5S, 16S, 23S)	44	4	42	42	56,6	141457	11
M. thermotolerans Sh4 (KMM 6837)														
JAPHQC000000000	3,400	3,346	3,313	3,313	54	1, 1, 1 (5S, 16S, 23S)	1, 1, 1 (5S, 16S, 23S)	47	4	33	33	56,6	152299	8
M. thermotolerans Sh3 (KMM 6836)														
JAPHQD000000000	3,337	3,286	3,248	3,248	51	1, 1, 1 (5S, 16S, 23S)	1, 1, 1 (5S, 16S, 23S)	44	4	38	38	56,6	249189	7

M. thermotolerans Sh2 (KMM 6835)													
JAPHQE000000000	3,386	3,335	3,291	3,291	51	1, 1, 1 (5S, 16S, 23S)	1, 1, 1 (5S, 16S, 23S)	44	4	44	44	56,4	141131	9
M. thermotolerans Sh1 (KMM 6242)													
JAPHQF000000000	3,366	3,315	3,276	3,276	51	1, 1, 1 (5S, 16S, 23S)	1, 1, 1 (5S, 16S, 23S)	44	4	39	39	56,5	181044	8
M. thermotolerans Sgm 25/1 (KMM 6834)													
JAPHQG000000000	3,374	3,323	3,289	3,289	51	1, 1, 1 (5S, 16S, 23S)	1, 1, 1 (5S, 16S, 23S)	44	4	34	34	56,5	153069	9
M. thermotolerans Sgf 25 (KMM 6262)													
JAPHQH000000000	3,366	3,315	3,276	3,276	51	1, 1, 1 (5S, 16S, 23S)	1, 1, 1 (5S, 16S, 23S)	44	4	39	39	56,6	146383	8

Table S4. Digital hybridization dDDH (d4%) for *Pseudoalteromonas* sp. B530 (= KMM 6841) and closely-related species *Pseudoalteromonas* spp. calculated by TYGS

Type Strain Genome Server								
**Query Strain**	**Subject Strain**	**dDDH (d0, in %)**	**C.I. (d0, in %)**	**dDDH (d4, in %)**	**C.I. (d4, in %)**	**dDDH (d6, in %)**	**C.I. (d6, in %)**	**G+C Content Difference (in %)**
'Pseudoalteromonas sp B530 contigs'	Pseudoalteromonas flavipulchra LMG 20361	73	[69.0 - 76.6]	63.5	[60.6 - 66.3]	73.6	[70.1 - 76.8]	0.14
'Pseudoalteromonas sp B530 contigs'	Pseudoalteromonas maricaloris LMG 19692	71.8	[67.9 - 75.5]	63.1	[60.2 - 65.9]	72.5	[69.0 - 75.7]	0.01
'Pseudoalteromonas sp B530 contigs'	Pseudoalteromonas piscicida ATCC 15057	74.7	[70.7 - 78.3]	61.2	[58.3 - 64.0]	74.5	[71.0 - 77.7]	0.12
'Pseudoalteromonas sp B530 contigs'	Pseudoalteromonas galatheae S4498T	67.1	[63.3 - 70.8]	48.3	[45.7 - 50.9]	64.4	[61.1 - 67.6]	0.11
'Pseudoalteromonas sp B530 contigs'	Pseudoalteromonas peptidolytica NBRC 101021	35	[31.6 - 38.5]	24	[21.7 - 26.5]	31.2	[28.2 - 34.3]	0.66
'Pseudoalteromonas sp B530 contigs'	Pseudoalteromonas tetraodonis DSM 9166	13.7	[10.9 - 17.0]	21	[18.7 - 23.4]	14	[11.6 - 16.8]	2.79
'Pseudoalteromonas sp B530 contigs'	Pseudoalteromonas espejiana ATCC 29659	13.7	[10.9 - 17.1]	20.9	[18.7 - 23.4]	14	[11.6 - 16.8]	2.8
'Pseudoalteromonas sp B530 contigs'	Pseudoalteromonas phenolica KCTC 12086	14.5	[11.7 - 17.9]	20.9	[18.7 - 23.3]	14.7	[12.3 - 17.6]	2.52

'Pseudoalteromonas sp B530 contigs'	Pseudoalteromonas issachenkonii KCTC 12958	13.7	[10.9 - 17.0]	20.9	[18.7 - 23.4]	14	[11.6 - 16.8]	2.81
'Pseudoalteromonas sp B530 contigs'	Pseudoalteromonas aliena DSM 16473	13.6	[10.8 - 16.9]	20.5	[18.3 - 22.9]	13.9	[11.5 - 16.7]	4.12
'Pseudoalteromonas sp B530 contigs'	Pseudoalteromonas spiralis DSM 16099	13.6	[10.8 - 16.9]	20.4	[18.1 - 22.8]	13.9	[11.5 - 16.7]	2.95
'Pseudoalteromonas sp B530 contigs'	Pseudoalteromonas carrageenovora ATCC 43555	13.7	[10.9 - 17.0]	20.4	[18.2 - 22.8]	13.9	[11.5 - 16.8]	3.68
'Pseudoalteromonas sp B530 contigs'	Pseudoalteromonas luteoviolacea DSM 6061	14.4	[11.5 - 17.8]	20.1	[17.9 - 22.5]	14.6	[12.1 - 17.4]	1.33
'Pseudoalteromonas sp B530 contigs'	Pseudoalteromonas caenipelagi JBTF-M23	14.8	[11.9 - 18.2]	19.8	[17.6 - 22.2]	14.9	[12.4 - 17.8]	1.12
'Pseudoalteromonas sp B530 contigs'	Pseudoalteromonas rhizosphaerae RA15	13.7	[10.9 - 17.0]	19.3	[17.1 - 21.7]	13.9	[11.5 - 16.7]	2.76

Table S5. Digital hybridization dDDH (d4%) for *Vibrio* sp. Sgm 5 (= KMM 6838), Sgm 22 (= KMM 6833), 14G-20 (KMM = 6839) and closely-related species *Vibrio* spp. calculated by TYGS

Type Strain Genome Server								
**Query Strain**	**Subject Strain**	**dDDH (d0, in %)**	**C.I. (d0, in %)**	**dDDH (d4, in %)**	**C.I. (d4, in %)**	**dDDH (d6, in %)**	**C.I. (d6, in %)**	**G+C Content Difference (in %)**
'Vibrio sp 14G-20 contigs'	'Vibrio sp Sgm22 contigs'	100	[100.0 - 100.0]	100	[99.9 - 100.0]	100	[100.0 - 100.0]	0
'Vibrio sp Sgm5 contigs'	Vibrio inhibens CECT 7692	88.3	[84.9 - 91.1]	79.4	[76.5 - 82.1]	89.6	[86.9 - 91.9]	0.09
'Vibrio sp Sgm5 contigs'	Vibrio jasicida CAIM 1864	86.8	[83.2 - 89.7]	78.9	[75.9 - 81.5]	88.3	[85.4 - 90.7]	0.07
'Vibrio sp Sgm5 contigs'	Vibrio hyugaensis 090810a	79.5	[75.6 - 83.0]	53.7	[51.0 - 56.4]	76.4	[73.0 - 79.6]	0.1
'Vibrio sp 14G-20 contigs'	Vibrio coralliirubri Corallo1	70.4	[66.5 - 74.1]	43.6	[41.1 - 46.2]	65.4	[62.0 - 68.6]	0.16
'Vibrio sp Sgm22 contigs'	Vibrio coralliirubri Corallo1	70.4	[66.5 - 74.0]	43.6	[41.1 - 46.2]	65.4	[62.0 - 68.6]	0.16
'Vibrio sp Sgm22 contigs'	Vibrio celticus Rd 8.15	70.4	[66.4 - 74.0]	43	[40.5 - 45.6]	65.2	[61.8 - 68.4]	0.01
'Vibrio sp 14G-20 contigs'	Vibrio celticus Rd 8.15	70.4	[66.4 - 74.0]	43	[40.5 - 45.6]	65.2	[61.8 - 68.4]	0.01
'Vibrio sp 14G-20 contigs'	Vibrio crassostreae LGP 7	64	[60.2 - 67.6]	40.2	[37.7 - 42.7]	59	[55.8 - 62.2]	0.11
'Vibrio sp Sgm22 contigs'	Vibrio crassostreae LGP 7	64	[60.2 - 67.6]	40.2	[37.7 - 42.7]	59	[55.7 - 62.1]	0.11
'Vibrio sp Sgm22 contigs'	Vibrio splendidus NCCB 53037	68	[64.2 - 71.7]	38.7	[36.2 - 41.2]	61.5	[58.2 - 64.7]	0.42

Table S6. Digital hybridization dDDH between the *M. thermotholerans* strains calculated by TYGS

Type Strain Genome Server								
**Query Strain**	**Subject Strain**	**dDDH (d0, in %)**	**C.I. (d0, in %)**	**dDDH (d4, in %)**	**C.I. (d4, in %)**	**dDDH (d6, in %)**	**C.I. (d6, in %)**	**G+C Content Difference (in %)**
'Microbulbifer thermotolerans 14G-22 contigs'	'Microbulbifer thermotolerans Sgm 25-1 contigs'	100	[100.0 - 100.0]	99.9	[99.8 - 100.0]	100	[100.0 - 100.0]	0.01
'Microbulbifer thermotolerans Sh 4 contigs'	'Microbulbifer thermotolerans Sh2 contigs'	96.5	[94.6 - 97.7]	95.7	[94.3 - 96.8]	97.8	[96.6 - 98.5]	0.17
'Microbulbifer thermotolerans Sh2 contigs'	'Microbulbifer thermotolerans Sh3 contigs'	95.3	[93.1 - 96.8]	95.7	[94.2 - 96.8]	97	[95.6 - 98.0]	0.15
'Microbulbifer thermotolerans Sh2 contigs'	Microbulbifer thermotolerans DSM 19189	96	[93.9 - 97.3]	95.6	[94.2 - 96.7]	97.4	[96.1 - 98.3]	0.07
'Microbulbifer thermotolerans Sh 4 contigs'	'Microbulbifer thermotolerans Sh3 contigs'	95.5	[93.3 - 97.0]	95.5	[94.1 - 96.7]	97.1	[95.7 - 98.1]	0.02
'Microbulbifer thermotolerans Sgm 25-1 contigs'	'Microbulbifer thermotolerans Sh1 contigs'	96.3	[94.3 - 97.6]	95.5	[94.0 - 96.6]	97.6	[96.4 - 98.4]	0.01
'Microbulbifer thermotolerans 14G-22 contigs'	'Microbulbifer thermotolerans Sh1 contigs'	96.3	[94.4 - 97.6]	95.5	[94.0 - 96.7]	97.6	[96.4 - 98.4]	0.02
'Microbulbifer thermotolerans 14G-22 contigs'	'Microbulbifer thermotolerans Sgf 25 contigs'	96.8	[95.1 - 98.0]	95.1	[93.5 - 96.3]	97.9	[96.8 - 98.7]	0.07

'Microbulbifer thermotolerans Sh3 contigs'	Microbulbifer thermotolerans DSM 19189	97.5	[96.0 - 98.5]	95.1	[93.6 - 96.3]	98.4	[97.4 - 99.0]	0.08
'Microbulbifer thermotolerans Sgf 25 contigs'	'Microbulbifer thermotolerans Sgm 25-1 contigs'	96.8	[95.1 - 98.0]	95	[93.4 - 96.2]	97.9	[96.8 - 98.7]	0.08
'Microbulbifer thermotolerans Sh 5 contigs'	'Microbulbifer thermotolerans Sh1 contigs'	96.3	[94.4 - 97.6]	95	[93.4 - 96.2]	97.6	[96.3 - 98.4]	0.12
'Microbulbifer thermotolerans Sh 4 contigs'	Microbulbifer thermotolerans DSM 19189	95.7	[93.6 - 97.1]	95	[93.4 - 96.2]	97.2	[95.8 - 98.1]	0.1
'Microbulbifer thermotolerans Sgm 25-1 contigs'	'Microbulbifer thermotolerans Sh 5 contigs'	95.5	[93.3 - 97.0]	94.9	[93.2 - 96.1]	97	[95.7 - 98.0]	0.11
'Microbulbifer thermotolerans 14G-22 contigs'	'Microbulbifer thermotolerans Sh 5 contigs'	95.6	[93.4 - 97.0]	94.9	[93.3 - 96.2]	97.1	[95.7 - 98.0]	0.09
'Microbulbifer thermotolerans Sgf 25 contigs'	'Microbulbifer thermotolerans Sh1 contigs'	96	[94.0 - 97.4]	94.8	[93.1 - 96.0]	97.4	[96.1 - 98.2]	0.1
'Microbulbifer thermotolerans Sgf 25 contigs'	'Microbulbifer thermotolerans Sh 5 contigs'	95.5	[93.4 - 97.0]	94.3	[92.6 - 95.7]	97	[95.6 - 98.0]	0.02
'Microbulbifer thermotolerans 14G-22 contigs'	'Microbulbifer thermotolerans Sh3 contigs'	93.9	[91.3 - 95.7]	93.6	[91.8 - 95.1]	95.8	[94.1 - 97.1]	0.1
'Microbulbifer thermotolerans Sgm 25-1 contigs'	'Microbulbifer thermotolerans Sh3 contigs'	93.9	[91.3 - 95.7]	93.6	[91.7 - 95.0]	95.8	[94.1 - 97.1]	0.11

'Microbulbifer thermotolerans Sh1 contigs'	'Microbulbifer thermotolerans Sh3 contigs'	95.5	[93.3 - 97.0]	93.5	[91.6 - 95.0]	96.9	[95.5 - 97.9]	0.12
'Microbulbifer thermotolerans 14G-22 contigs'	'Microbulbifer thermotolerans Sh 4 contigs'	94.3	[91.9 - 96.1]	93.5	[91.6 - 94.9]	96.1	[94.4 - 97.3]	0.12
'Microbulbifer thermotolerans Sgf 25 contigs'	'Microbulbifer thermotolerans Sh3 contigs'	95.7	[93.5 - 97.1]	93.5	[91.6 - 94.9]	97	[95.6 - 98.0]	0.03
'Microbulbifer thermotolerans Sgf 25 contigs'	'Microbulbifer thermotolerans Sh2 contigs'	95.3	[93.1 - 96.9]	93.5	[91.6 - 94.9]	96.8	[95.3 - 97.8]	0.12
'Microbulbifer thermotolerans Sgm 25-1 contigs'	'Microbulbifer thermotolerans Sh 4 contigs'	94.4	[91.9 - 96.1]	93.4	[91.5 - 94.9]	96.1	[94.4 - 97.3]	0.13
'Microbulbifer thermotolerans Sh 4 contigs'	'Microbulbifer thermotolerans Sh1 contigs'	95.8	[93.7 - 97.2]	93.4	[91.5 - 94.9]	97.1	[95.7 - 98.0]	0.14
'Microbulbifer thermotolerans Sgf 25 contigs'	'Microbulbifer thermotolerans Sh 4 contigs'	94.7	[92.4 - 96.4]	93.3	[91.4 - 94.8]	96.4	[94.8 - 97.5]	0.05
'Microbulbifer thermotolerans Sh 5 contigs'	'Microbulbifer thermotolerans Sh3 contigs'	94.6	[92.2 - 96.3]	93.3	[91.4 - 94.8]	96.3	[94.6 - 97.4]	0
'Microbulbifer thermotolerans Sh1 contigs'	'Microbulbifer thermotolerans Sh2 contigs'	95.6	[93.5 - 97.1]	93.2	[91.3 - 94.7]	97	[95.5 - 97.9]	0.02
'Microbulbifer thermotolerans 14G-22 contigs'	'Microbulbifer thermotolerans Sh2 contigs'	94	[91.5 - 95.9]	93.2	[91.3 - 94.7]	95.9	[94.2 - 97.1]	0.05

'Microbulbifer thermotolerans Sgf 25 contigs'	Microbulbifer thermotolerans DSM 19189	95.3	[93.1 - 96.9]	93.2	[91.3 - 94.7]	96.8	[95.3 - 97.8]	0.05
'Microbulbifer thermotolerans Sgm 25-1 contigs'	'Microbulbifer thermotolerans Sh2 contigs'	94.1	[91.6 - 95.9]	93.1	[91.2 - 94.6]	95.9	[94.2 - 97.1]	0.03
'Microbulbifer thermotolerans Sh 4 contigs'	'Microbulbifer thermotolerans Sh 5 contigs'	94.3	[91.8 - 96.1]	92.9	[91.0 - 94.5]	96	[94.3 - 97.2]	0.02
'Microbulbifer thermotolerans Sh 5 contigs'	'Microbulbifer thermotolerans Sh2 contigs'	95	[92.6 - 96.6]	92.6	[90.7 - 94.2]	96.4	[94.9 - 97.5]	0.14
'Microbulbifer thermotolerans 14G-22 contigs'	Microbulbifer thermotolerans DSM 19189	92.9	[90.1 - 94.9]	92.6	[90.6 - 94.2]	95	[93.1 - 96.4]	0.02
'Microbulbifer thermotolerans Sh1 contigs'	Microbulbifer thermotolerans DSM 19189	95.4	[93.2 - 96.9]	92.5	[90.5 - 94.1]	96.7	[95.2 - 97.8]	0.04
'Microbulbifer thermotolerans Sgm 25-1 contigs'	Microbulbifer thermotolerans DSM 19189	92.9	[90.1 - 94.9]	92.5	[90.5 - 94.2]	95	[93.1 - 96.4]	0.03
'Microbulbifer thermotolerans Sh 5 contigs'	Microbulbifer thermotolerans DSM 19189	94.5	[92.1 - 96.2]	92.4	[90.4 - 94.0]	96.1	[94.5 - 97.3]	0.08

Table S7. Functional annotation of chitinase genes of *M. thermotolerans*

Strain	Domain organization according to CAZy and dbCAN (data from 05 Dec 2022)	Database
M. thermotolerans DAU221 (CP014864)	*GH18:*<br>CBM5, CBM73, GH18 (AMX02245)<br>CBM5, CBM73, GH18 (AMX03031)<br>CBM73, CBM5, GH18 (AMX03032)<br>CBM73, GH18, CBM5 (AMX03073)<br>*(Cazy)*	CAZy<br>http://www.cazy.org
	AA10:<br>AA10, CBM73 (AMX02527)<br>AA10, CBM2 (AMX03411)<br>*(Cazy)*	
M. thermotolerans JCM 14709^T (SAMN05660479)	*GH18:*<br>CBM5, CBM73, GH18 (NZ_FOKT01000001.1|5626|GCA_900112305.1_02813|530561..532180)<br>CBM5, CBM73, GH18 (NZ_FOKT01000006.1|1624|GCA_900112305.1_00812|complement(23695..26805)<br>CBM5, CBM73, GH18 (NZ_FOKT01000006.1|1728|GCA_900112305.1_00864|87071..88885)<br>CBM5, CBM73, GH18 (NZ_FOKT01000006.1|1732|GCA_900112305.1_00866|90373..91902)<br>*(dbCAN)*	Ezbiocloud<br>https://www.ezbiocloud.net
	AA10:<br>AA10, CBM73 (NZ_FOKT01000002.1|3218|GCA_900112305.1_01609|complement(101220..102650)<br>AA10, CBM2 (NZ_FOKT01000005.1|122|GCA_900112305.1_00061|66068..67666)<br>*(dbCAN)*	
M. thermotolerans Sgf 25 (JAPHQH000000000)	*GH18:*<br>CBM5, CBM73, GH18 (fig|252514.29.peg.1292)<br>CBM5, CBM73, GH18 (fig|252514.29.peg.3182)<br>CBM5, CBM73, GH18 (fig|252514.29.peg.3184)<br>CBM5, CBM73, GH18 (fig|252514.29.peg.3236)<br>*(dbCAN)*	NCBI (GeneBank), bioproject № PRJNA898858 «Chitinolytic marine bacteria from the Collection of Marine Microorganisms (KMM)»<br>https://www.ncbi.nlm.nih.gov
	AA10:<br>AA10, CBM73 (fig|252514.29.peg.2913)<br>AA10, CBM2 (fig|252514.29.peg.486)<br>AA10, CBM2 (fig|252514.29.peg.488)<br>*(dbCAN)*	

M. thermotolerans Sgm 25/1 (JAPHQG000000000)	*GH18:*<br>CBM5, CBM73, GH18 (fig|252514.22.peg.2443)<br>CBM5, CBM73, GH18 (fig|252514.22.peg.3144)<br>CBM5, CBM73, GH18 (fig|252514.22.peg.3196)<br>CBM5, CBM73, GH18 (fig|252514.22.peg.3198)<br>*(dbCAN)*	NCBI (GeneBank), bioproject № PRJNA898858 «Chitinolytic marine bacteria from the Collection of Marine Microorganisms (KMM)» https://www.ncbi.nlm.nih.gov
	AA10:<br>AA10+CBM2 (fig|252514.22.peg.1585)<br>AA10, CBM2 (fig|252514.22.peg.1587)<br>AA10, CBM73 (fig|252514.22.peg.666)<br>*(dbCAN)*	
M. thermotolerans Sh 1 (JAPHQF000000000)	*GH18:*<br>CBM5, CBM73, GH18 (fig|252514.28.peg.2696)<br>CBM5, CBM73, GH18 (fig|252514.28.peg.413)<br>CBM5, CBM73, GH18 (fig|252514.28.peg.466)<br>CBM5, CBM73, GH18 (fig|252514.28.peg.468)<br>*(dbCAN)*	NCBI (GeneBank), bioproject № PRJNA898858 «Chitinolytic marine bacteria from the Collection of Marine Microorganisms (KMM)» https://www.ncbi.nlm.nih.gov
	AA10:<br>AA10, CBM73 (fig|252514.28.peg.1107)<br>AA10, CBM2 (fig|252514.28.peg.545)<br>AA10, CBM2 (fig|252514.28.peg.547)<br>*(dbCAN)*	
M. thermotolerans Sh 2 (JAPHQE000000000)	*GH18:*<br>CBM5, CBM73, GH18 (fig|252514.27.peg.3368)<br>CBM5, CBM73, GH18 (fig|252514.27.peg.368)<br>CBM5, CBM73, GH18 (fig|252514.27.peg.370)<br>CBM5, CBM73, GH18 (fig|252514.27.peg.424)<br>*(dbCAN)*	NCBI (GeneBank), bioproject № PRJNA898858 «Chitinolytic marine bacteria from the Collection of Marine Microorganisms (KMM)» https://www.ncbi.nlm.nih.gov
	AA10:<br>AA10, CBM73 (fig|252514.27.peg.1799)<br>AA10, CBM2 (fig|252514.27.peg.563)<br>AA10, CBM2 (fig|252514.27.peg.565)<br>*(dbCAN)*	
M. thermotolerans Sh 3 (JAPHQD000000000)	*GH18:*<br>CBM5, CBM73, GH18 (fig|252514.26.peg.1535)<br>CBM5, CBM73, GH18 (fig|252514.26.peg.329)<br>CBM5, CBM73, GH18 (fig|252514.26.peg.383)<br>CBM5, CBM73, GH18 (fig|252514.26.peg.385)<br>*(dbCAN)*	NCBI (GeneBank), bioproject № PRJNA898858 «Chitinolytic marine bacteria from the Collection of Marine Microorganisms (KMM)» https://www.ncbi.nlm.nih.gov

	AA10:<br>AA10, CBM73 (fig|252514.26.peg.3092)<br>AA10, CBM2 (fig|252514.26.peg.37)<br>*(dbCAN)*	
M. thermotolerans Sh 4 (JAPHQC000000000)	*GH18:*<br>CBM5, CBM73, GH18 (fig|252514.21.peg.1112)<br>CBM5, CBM73, GH18 (fig|252514.21.peg.1114)<br>CBM5, CBM73, GH18 (fig|252514.21.peg.1526)<br>CBM5, CBM73, GH18 (fig|252514.21.peg.1999)<br>*(dbCAN)*	NCBI (GeneBank), bioproject № PRJNA898858 «Chitinolytic marine bacteria from the Collection of Marine Microorganisms (KMM)» https://www.ncbi.nlm.nih.gov
	AA10:<br>AA10, CBM73 (fig|252514.21.peg.2707)<br>AA10, CBM2 (fig|252514.21.peg.3431)<br>*(dbCAN)*	
M. thermotolerans Sh 5 (JAPHQB000000000)	*GH18:*<br>CBM5, CBM73, GH18 (fig|252514.23.peg.2705)<br>CBM5, CBM73, GH18 (fig|252514.23.peg.434)<br>CBM5, CBM73, GH18 (fig|252514.23.peg.436)<br>CBM5, CBM73, GH18 (fig|252514.23.peg.490)<br>*(dbCAN)*	NCBI (GeneBank), bioproject № PRJNA898858 «Chitinolytic marine bacteria from the Collection of Marine Microorganisms (KMM)» https://www.ncbi.nlm.nih.gov
	AA10:<br>AA10, CBM2 (fig|252514.23.peg.1527)<br>AA10, CBM2 (fig|252514.23.peg.1529)<br>AA10, CBM73 (fig|252514.23.peg.2094)<br>*(dbCAN)*	
