# Supplementary figures and images for: Chitinolytic and Fungicidal Potential of the Marine Bacterial Strains Habituating Pacific Ocean Regions
Source: Microorganisms. 2023 Sep 8;11(9):2255. doi: 10.3390/microorganisms11092255 (PMC10535946; doi:10.3390/microorganisms11092255)

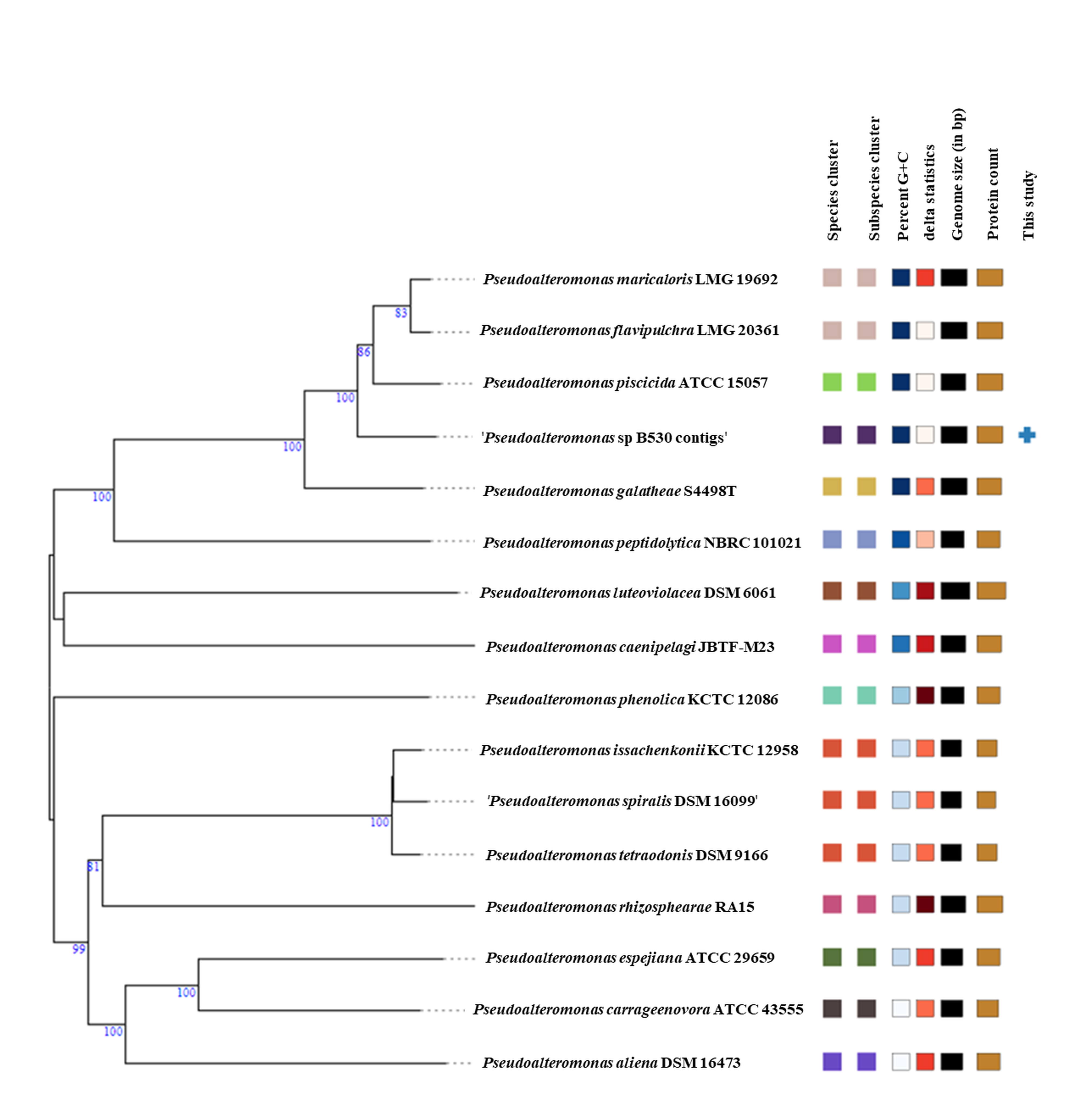

Supplement: Supplementary file 1 [file microorganisms-11-02255-s001.zip › Figure S1.png]

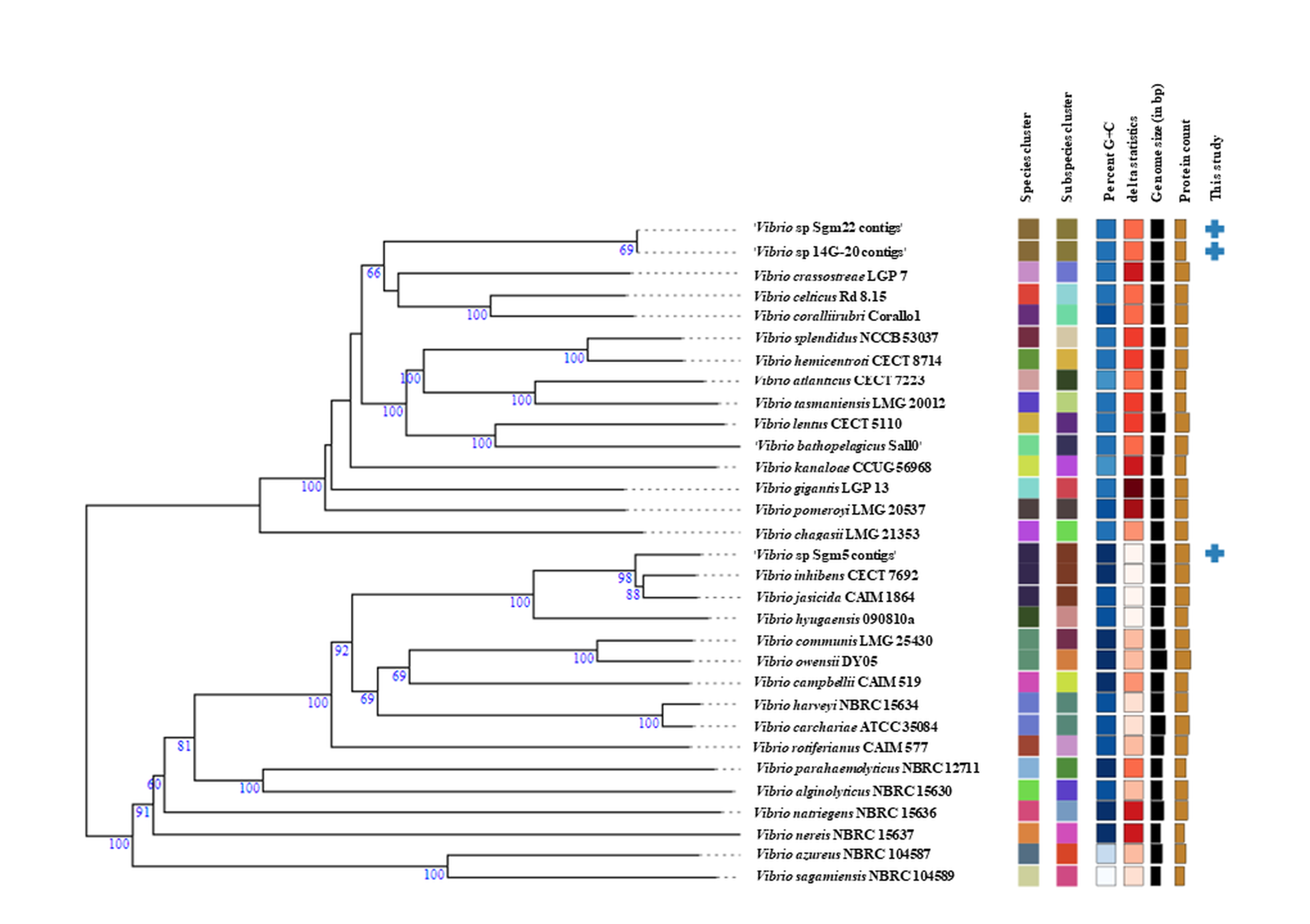

Supplement: Supplementary file 1 [file microorganisms-11-02255-s001.zip › Figure S2.png]

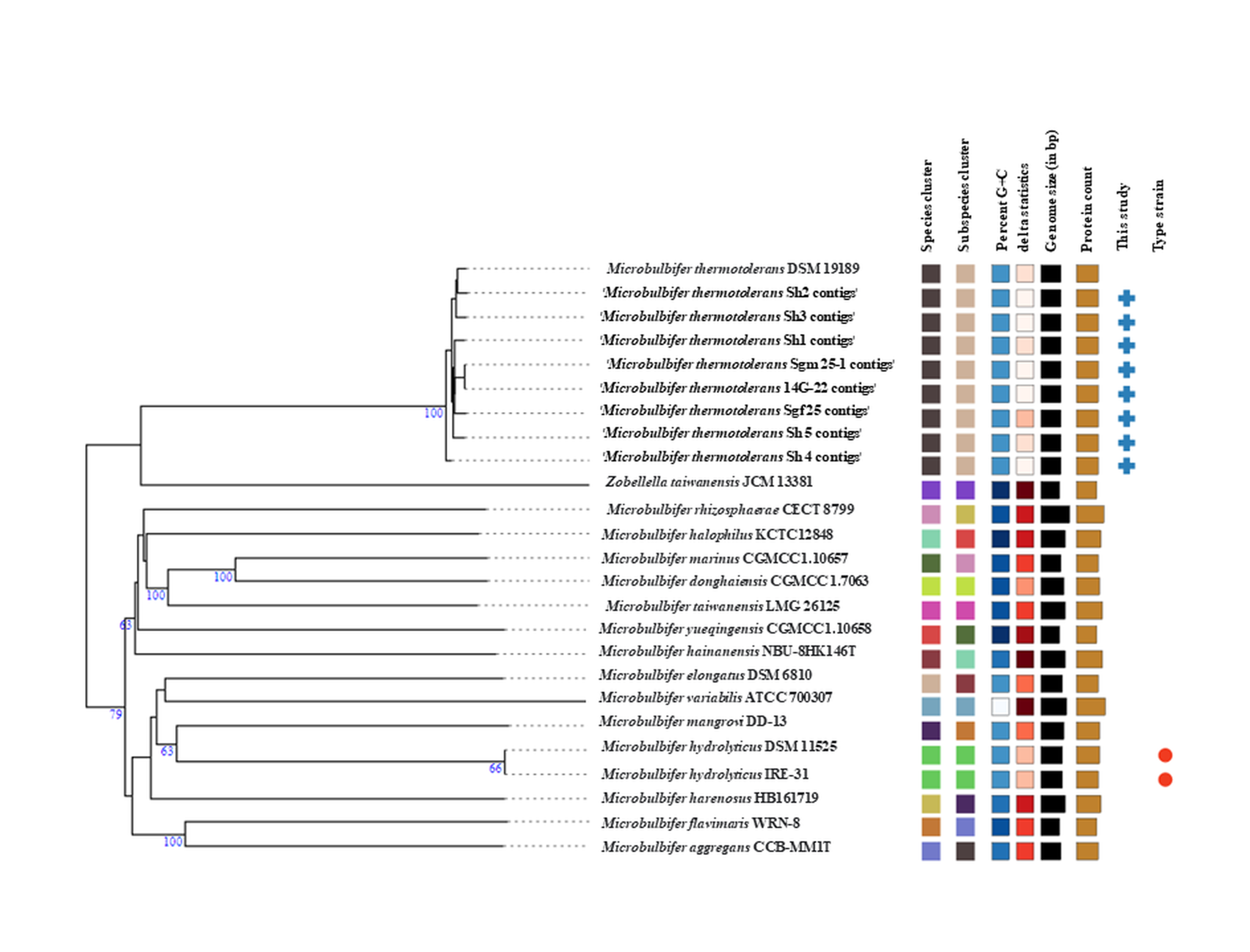

Supplement: Supplementary file 1 [file microorganisms-11-02255-s001.zip › Figure S3.png]

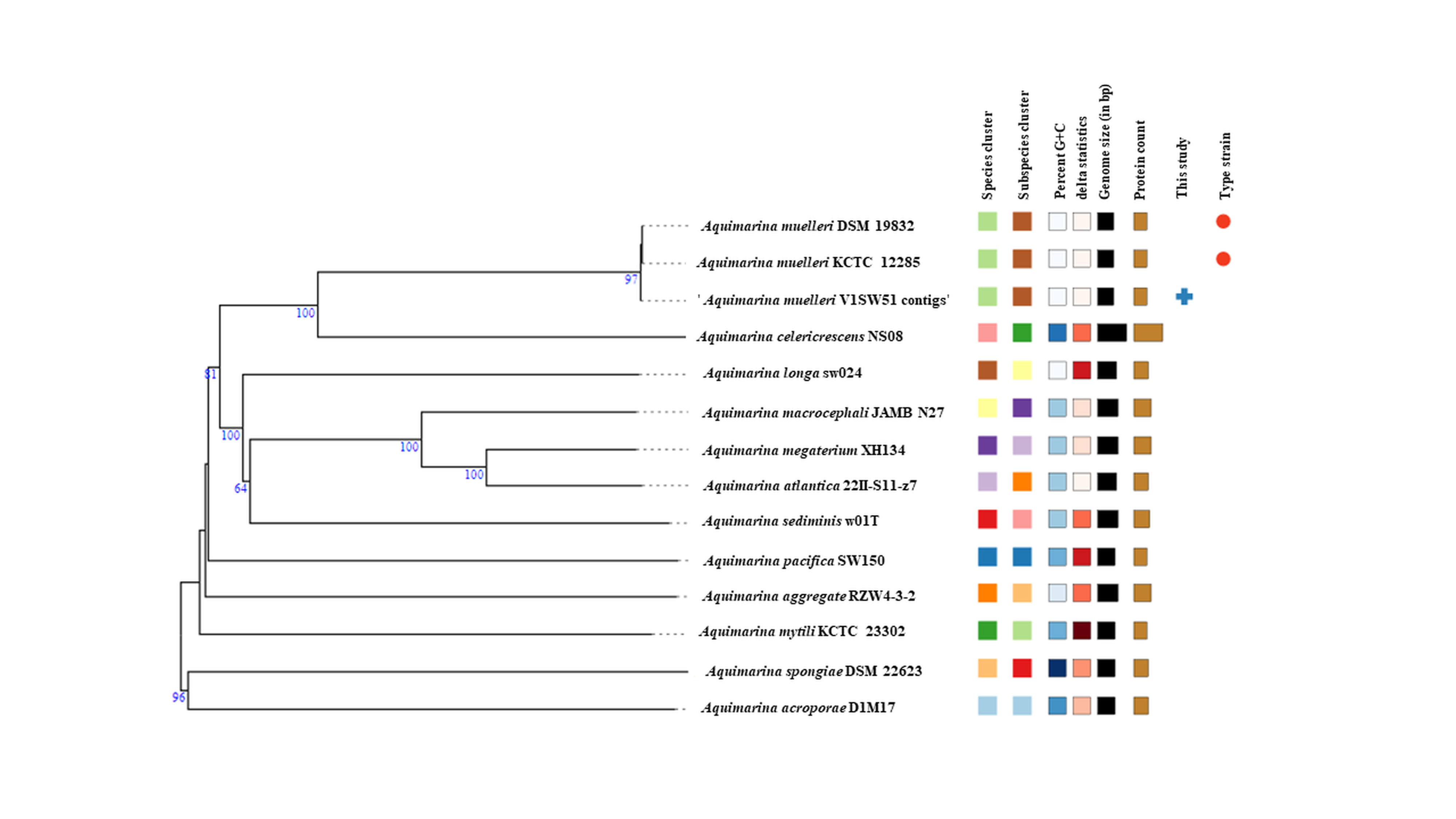

Supplement: Supplementary file 1 [file microorganisms-11-02255-s001.zip › Figure S4.png]
